# Free Saturated Oxo Fatty Acids (SOFAs) and Ricinoleic Acid in Milk Determined by a Liquid Chromatography-High-Resolution Mass Spectrometry (LC-HRMS) Method

**DOI:** 10.3390/metabo11010046

**Published:** 2021-01-11

**Authors:** Maroula G. Kokotou, Charikleia S. Batsika, Christiana Mantzourani, George Kokotos

**Affiliations:** Department of Chemistry, National and Kapodistrian University of Athens, 15771 Athens, Greece; mkokotou@chem.uoa.gr (M.G.K.); cbatsika@chem.uoa.gr (C.S.B.); chrmantz@chem.uoa.gr (C.M.)

**Keywords:** determination, high-resolution mass spectrometry, liquid chromatography, milk, oxo fatty acids

## Abstract

Oxidized saturated fatty acids, containing a hydroxyl or an oxo functionality, have attracted little attention so far. Recent studies have shown that saturated hydroxy fatty acids, which exhibit cancer cell growth inhibition and may suppress β-cell apoptosis, are present in milk. Herein, we present the application of a liquid chromatography-high-resolution mass spectrometry (LC-HRMS) method for the detection and quantification of various saturated oxo fatty acids (SOFAs) previously unrecognized in milk. This robust and rapid analytical method, which involves simple sample preparation and a single 10-min run, revealed the presence of families of oxostearic acids (OSAs) and oxopalmitic acids (OPAs) in milk. 8OSA, 9OSA, 7OSA, 10OSA and 10OPA were found to be the most abundant SOFAs in both cow and goat milk. Higher contents of SOFAs were found in cow milk in comparison to goat milk. Together with SOFAs, ricinoleic acid, which is isobaric to OSA, was detected and quantified in all milk samples, following a “suspect” HRMS analysis approach. This unique natural fatty acid, which is the main component (>90%) of castor oil triglycerides, was estimated at mean content values of 534.3 ± 6.0 μg/mL and 460 ± 8.1 μg/mL in cow and goat milk samples, respectively.

## 1. Introduction

Milk fat is one of the most complex natural fats because of the presence of more than 400 different fatty acids (FAs) [1,2,3]. These FAs vary in carbon chain length (from 4 to 26 carbons), including FAs with either an even or an odd number of carbon atoms, in a straight or a branched chain. They also vary in the degree of unsaturation, including geometrical isomers, with double bonds in mainly cis but also in trans configuration. Both the physicochemical and sensory properties of milk and the nutritional quality of milk and dairy fat are mainly dependent on its FA composition. Fourteen FAs are present in milk fat at concentrations above 1%; however, all the rest of the FAs exist in very low quantities (less than 0.01% of the total) [4]. Little attention has been paid so far to oxygenated types of saturated FAs. Thus, the available data reported on the occurrence of saturated hydroxy fatty acids (SHFAs) and saturated oxo (keto) fatty acids (SOFAs) in milk are scarce.

Although the presence of 2-hydroxy and 3-hydroxy FAs in milk and dairy products was reported more than 10 years ago [5,6], only one report demonstrated the existence of two other SHFAs, 10-hydroxystearic acid and 8-hydroxypalmitic acid, in milk [7]. This year, we reported the identification in milk of new families of previously unrecognized SHFAs, namely hydroxystearic acids (HSAs) and hydroxypalmitic acids (HPAs), which exhibit cell growth inhibitory activity and the ability to inhibit cytokine-induced β-cell apoptosis [8]. 7-(*R*)-Hydroxystearic acid exhibited the highest potency both in cell growth inhibition and in suppressing β-cell death [8]. A liquid chromatography-high-resolution mass spectrometry (LC-HRMS) method has been developed for the determination of such important bioactive SHFAs in cow and goat milk [9]. Most recently, a strategy for the annotation of SHFAs based on the integration of chromatographic retention rules and MS^2^ fragmentation patterns has been reported, and SHFAs have been detected in honey, human sera and rice seedlings [10].

SOFAs constitute another class of oxidized lipids, unexplored in foods so far. A few examples of the presence of such FAs in milk have been reported. Oxostearic acids (OSAs) were found to exist in milk in 1963 [11], and 16-ketostearic acid was identified as the most predominant among eight different OSAs in rumen [12]. In recent years, 10-ketostearic acid and 8-ketopalmitic acid have been detected in milk by gas chromatography-mass spectrometry (GC-MS) [7].

Particular milk fatty acids have been positively correlated with human health [13], and, as a consequence, the development of reliable and fast analytical methods for the determination of total and free FAs in milk is of great importance. A variety of methods exist for the determination of FAs in milk and dairy products in general [14]; however, the most common approach for the quantification of FAs in dairy products involves the use of gas chromatography flame ionization detection (GC-FID) and the conversion of FAs into the corresponding methyl esters [15,16]. Recently, analytical methods for the determination of either total or free FAs have employed HPLC-electrospray ionization (ESI)-Q-time of flight (ToF) [17] or LC-HRMS approaches [18].

The aim of the present work was the development of a method for the determination of a series of SOFAs in milk in order to get a comprehensive picture of the presence of such trace FAs in milk. Herein, we describe an LC-HRMS method for the direct determination of SOFAs, applying a simple and mild extraction protocol for sample preparation and avoiding any prior derivatization step. A family of OSAs and oxopalmitic acids (OPAs) was identified and quantified in both cow and goat milk after applying this method. In addition, for the first time, ricinoleic acid was detected and quantified in all milk samples.

## 2. Results

### 2.1. LC-ESI-MS Data

In the present study, thirteen SOFAs were used. Six of them were regioisomers of oxopalmitic acid (14-oxopalmitic acid (14OPA), 10-oxopalmitic acid (10OPA), 9-oxopalmitic acid (9OPA), 8-oxopalmitic acid (8OPA), 7-oxopalmitic acid (7OPA) and 6-oxopalmitic acid (6OPA)), and seven were regioisomers of oxostearic acid (16-oxostearic acid (16OSA), 12-oxostearic acid (12OSA), 10-oxostearic acid (10OSA), 9-oxostearic acid (9OSA), 8-oxostearic acid (8OSA), 7-oxostearic acid (7OSA) and 6-oxostearic acid (6OSA)). ESI negative mode was used for recording the HRMS spectra of the SOFAs studied. The structures, the exact masses of the deprotonated molecules and the retention times observed for each one of the SOFAs in the chromatographic method are summarized in Table 1.

The MS/MS spectra of all the 13 SOFAs in negative ESI mode are shown in Figure S1 (Supplementary Materials). In all cases, a fragment generated from the loss of CO_2_ was observed. The exact mass of this fragment is *m*/*z* 225.2224 in the series of OPAs and *m*/*z* 253.2537 in the series of OSAs.

### 2.2. Method Validation

The limits of detection (LOD) and quantification (LOQ), together with the calibration curve data, are summarized in Table 2. Good linearity values were observed for all the analytes (R^2^ > 0.99). The limits of detection varied from 0.3 to 0.8 ng/mL, while the limits of quantification varied from 1.0 to 2.4 ng/mL.

The sample preparation procedure consisted of the addition of methanol for protein precipitation, followed by centrifugation. Then, the supernatant was used for analysis. The guidelines of the EU Commission decision 202/657/EC were followed to verify accuracy and precision. Milk samples were spiked at three different concentration levels with three replicates for each fortification level. Satisfactory recoveries, ranging from 73.4 to 90.1 for the low spike level, from 73.9 to 99.5 for the medium spike level and from 81.7 to 112.0 for the high spike level (Table 3), indicated the accuracy of the proposed method. Precision was investigated by means of the relative standard deviation (RSD, %). The RSD values that were obtained for intraday (RSDr) and interday (RSD_R_) variations ranged from 0.1 to 9.3 and from 0.8 to 11.5, respectively, depending on the SOFAs (Table 3). The matrix factor (MF) values are also included in Table 3. Such a value was calculated as the ratio of the peak response in the presence of a matrix to the peak response in pure solvent. MF values < 1 indicate signal suppression in the samples, while MF values > 1 indicate signal enhancement.

### 2.3. Liquid Chromatography

**Figure 1:**
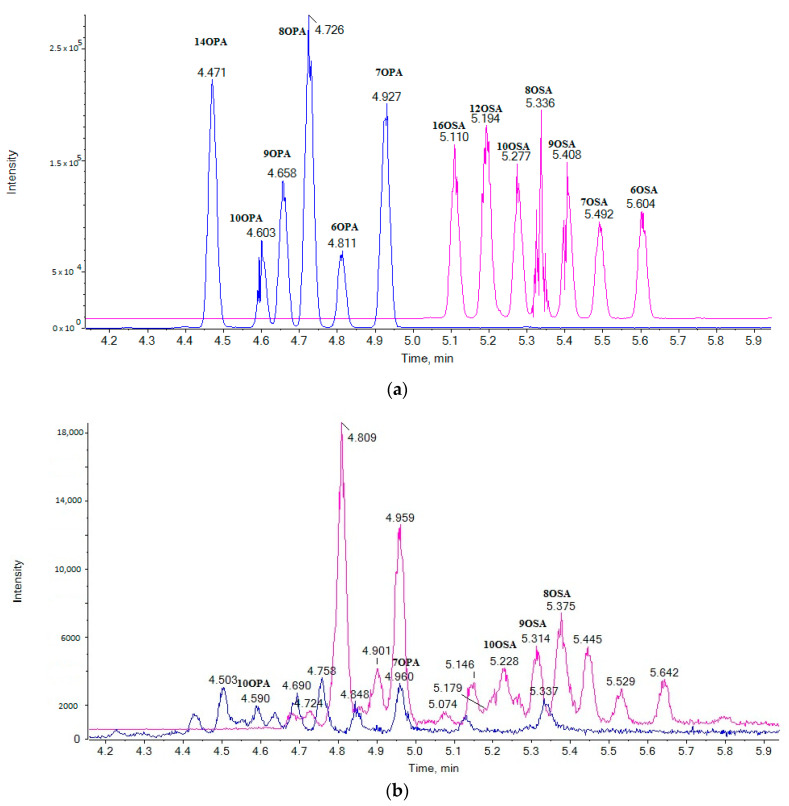
Extracted ion chromatograms (EICs) of oxopalmitic acids (OPAs) (blue line, exact mass *m*/*z* 269.2122) and oxostearic acids (OSAs) (magenta line, exact mass *m*/*z* 297.2435) in a standard solution (0.5 µg/mL) (**a**) and in a representative cow milk sample (**b**).

After performing experiments on chromatographic conditions (run time and gradient used), a rapid LC-HRMS method was applied permitting the simultaneous determination of 13 SOFAs in milk in a 10-min run. The extracted ion chromatograms (EICs) of the analytes in a standard solution (0.5 µg/mL) (a) and in a cow milk sample (b) are shown in Figure 1.

### 2.4. Suspect Analysis

It is known that the retention of small-molecule solutes on the C18 stationary phase in reversed-phase chomatography depends on the hydrophobic interactions between the solute and the stationary phase [19]. The length of the alkyl chain of SOFAs is the predominant factor in determining their retention. As a consequence, retention time increases with the increase of the carbon atom chain length, and OPAs (C16) are eluted first in comparison to OSAs (C18). Thus, it seems that the position of the oxo functionality influences the retention of the various regioisomers within a given group of solutes having the same alkyl chain length. When the oxo functionality is closest to the carboxyl group, the retention time for a solute is the highest. As shown in Figure 1a, the elution follows the order 14OPA, 10OPA, 9OPA, 8OPA, 7OPA and 6OPA for the OPA derivatives and the order 16OSA, 12OSA, 10OSA, 9OSA, 8OSA, 7OSA and 6OSA for the OSA derivatives.

Although the presence of 8OSA, 9OSA and 10OSA was clearly observed in a cow milk sample (as shown in Figure 1b) the most intense peak was observed at 4.81 min, and this peak was unlikely to correspond to an OSA regioisomer. Taking into consideration that an exact mass of 297.2435 may correspond either to a saturated oxo C18 carboxylic acid or to an isobaric monounsaturated hydroxy C18 carboxylic acid, we thought that the peak eluted at 4.81 min may have corresponded to a naturally occurring monounsaturated hydroxy C18 carboxylic acid, possibly to ricinoleic acid (RA) (12-hydroxy-9-cis-octadecenoic acid), rather than to an OSA. RA is not expected to be synthesized in rumen; however, it could be found in milk depending on the type of animal feeding. As a matter of fact, about 90% of the fatty acid content of castor oil is the triglyceride formed from RA, and castor oil may supplement cow feeding [20,21].

**Figure 2:**
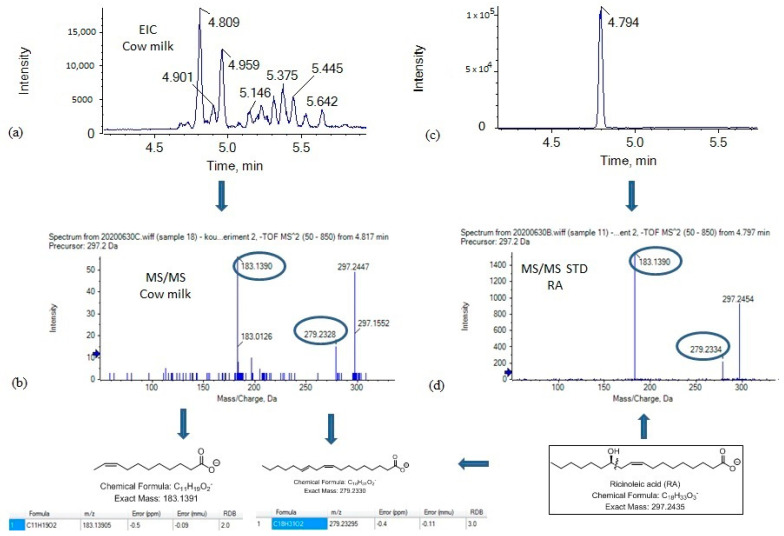
Mass spectrometry analysis of ricinoleic acid in cow milk. (**a**) EIC of OSAs (*m*/*z* 297.2435) in a representative cow milk sample: (**b**) MS/MS spectrum of the precursor ion [M − H]^−^; (**c**) EIC of standard ricinoleic acid; (**d**) MS/MS spectrum of standard ricinoleic acid.

The MS/MS spectrum of the precursor ion [M − H]^−^ (*m*/*z* 297.2435), which corresponds to the analyte eluted at 4.81 min (Figure 2a), is depicted in Figure 2b. Two main fragments were observed at *m*/*z* 183.1390 and *m*/*z* 279.2328. The first one corresponds to C_11_H_16_O_2_^−^ (exact mass 183.1391) and may be attributed to an ion generated by the α-cleavage of RA, while the second one corresponds to C_18_H_31_O_2_^−^ (exact mass 279.2329) and may be attributed to an ion generated by loss of H_2_O. Such a fragmentation matches that of standard RA, the MS/MS spectrum of which is depicted in Figure 2d. In addition, the retention time for RA, under the same chromatographic conditions, was found to be 4.79 min (Figure 2c). Thus, we propose that the most intense peak observed at 4.81 min is attributable to RA.

### 2.5. Analysis of Samples

**Figure 3:**
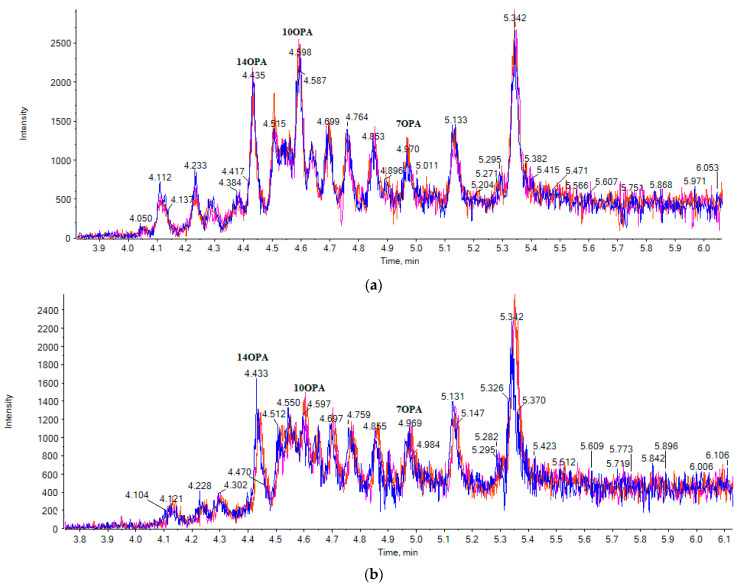
EICs of the multisample analysis for OPAs (exact mass *m*/*z* 269.2122) in three cow milk samples (**a**) and in three goat milk samples (**b**).

**Figure 4:**
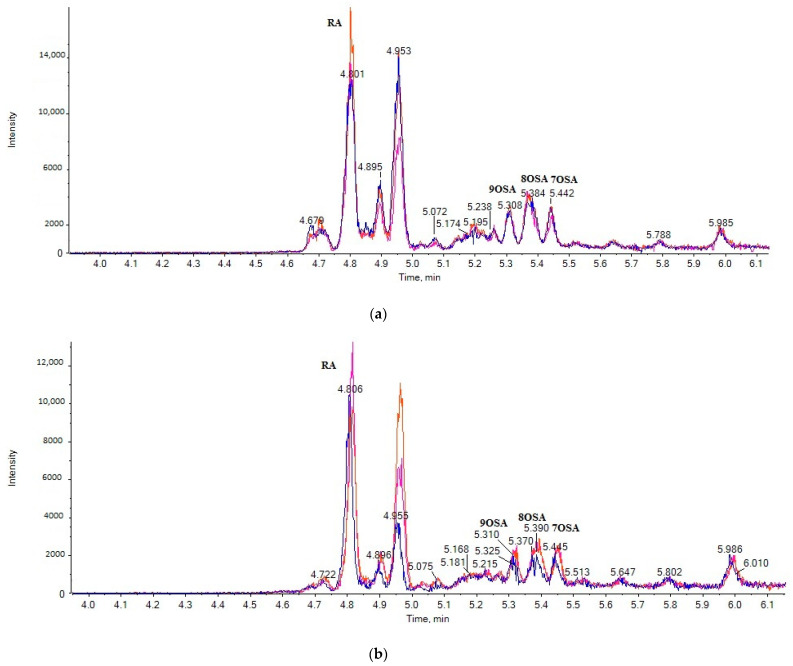
EICs of the multisample analysis for OSAs (exact mass *m*/*z* 297.2435) in three cow milk samples (**a**) and in three goat milk samples (**b**).

Twelve cow milk samples and eight goat milk samples, purchased from the local market, were analyzed. The multisample analysis data of three cow milk samples (a) and three goat milk samples (b) are shown in Figure 3 and Figure 4. The contents of the 14 analytes in milk samples (in triplicate) are summarized in Table 4 and expressed as μg of SOFA or RA per mL of fresh milk. The integration of the peaks was performed manually using MultiQuant 3.0.2 (ABSciex, Darmstadt, Germany). Manual baseline was employed for quantification of the peaks, as suggested by Hill et al., in difficult situations [22]. The same parameters were used for integration of the samples each time.

In cow milk, 10OPA was found to be the most abundant among OPAs (76.7 ± 7.2 μg/mL), followed by 7OPA (43.6 ± 5.2 μg/mL), while the contents of the rest of the OPAs varied from 12.5 ± 4.1 μg/mL to 23.3 ± 5.6 μg/mL. 10OPA was also the most abundant in goat milk (41.9 ± 5.6 μg/mL), while the contents of the rest of the OPAs varied from 17.3 ± 4.1 μg/mL to as little as 1.6 ± 2.9 μg/mL.

Among the OSAs, 8OSA, 9OSA, 7OSA and 10OSA were estimated at levels of 96.9 ± 7.2 μg/mL, 89.0 ± 6.5 μg/mL, 66.8 ± 6.5 μg/mL and 47.6 ± 4.6 μg/mL, respectively, in cow milk. The contents of the rest of the OSAs varied from 17.4 ± 5.1 μg/mL to 6.5 ± 6.1 μg/mL, while 6OSA was absent from all cow milk samples. In goat milk, 9OSA was found at the highest level of 66.9 ± 7.1 μg/mL. The contents of the other OSAs were estimated at mean values of 45.6 ± 8.0 μg/mL to 6.8 ± 5.3 μg/mL, but 6OSA and 16OSA were absent from all goat milk samples.

RA was determined at much higher levels in both cow and goat milk. Its levels in cow milk varied from 620.2 to 181.7 μg/mL, with a mean value of 534.3 ± 6.0 μg/mL. In goat milk, RA was found at a mean value of 460.4 ± 8.1 μg/mL, with levels varying from 411.2 to 231.1 μg/mL. To our knowledge, this is the first time that the levels of RA in real fresh milk samples, either cow or goat, have been reported. RA is a unique unsaturated fatty acid, which can be found only in castor oil and not in other vegetable oils. Thus, its presence in milk leads to the conclusion that castor oil has been used as an animal feeding supplement.

In the present work, we have quantified six different OSAs (7OSA, 8OSA, 9OSA, 10OSA, 12OSA and 16OSA) in cow milk and five different OSAs in goat milk (7OSA, 8OSA, 9OSA, 10OSA and 12OSA). Only the presence of 10OSA was reported in the past, in milk from animals fed olive oil (up to 1.5%) and from those fed long-chain ω-3 FA-enriched diets (0.5–1.0%) [7]. 10OSA may be generated in the rumen from oleic acid, as demonstrated in previous reports [23,24,25,26], and then it may be transferred to the mammary gland [7]. Furthermore, the microbial activity in the rumen has been shown to metabolize RA to produce 12OSA via the corresponding 12-hydroxystearic acid, and then 12OSA may be excreted in milk [27]. We have also quantified six different OPAs (6OPA, 7OPA, 8OPA, 9OPA, 10OPA and 14OPA) in both cow and goat milk. Only 8OPA was previously detected in milk [7], and its presence was attributed to fatty acid β-oxidation in the tissues [7]. This means that a multistep process leads to a FA that is two carbons shorter than the original via the breakdown of the initial aliphatic chain close to the carboxyl group. Upon such a two-atom carbon chain length reduction via β-oxidation, 8OPA may be generated from 10OSA, and 10OPA may be generated from 12OSA.

## 3. Materials and Methods

### 3.1. Chemicals and Reagents

LC-MS analytical grade solvents were used. Acetonitrile was purchased from Carlo Erba (Chaussée du Vexin, Val De Reuil, France), isopropanol and methanol from Fisher Scientific (Laughborough, UK) and formic acid 98–100% from Chem-Lab (Zedelgem, Belgium). RA was commercially available from Cayman Chemical (Ann Arbor, MI, USA). 14OPA, 10OPA, 9OPA, 8OPA, 7OPA, 6OPA, 16OSA, 12OSA, 10OSA, 9OSA, 8OSA, 7OSA and 6OSA were synthesized at the Laboratory of Organic Chemistry, National and Kapodistrian University of Athens, and the details of their synthesis will be published elsewhere. The standards were characterized by ^1^H and ^13^C NMR spectroscopy and HRMS. Their purity was studied by LC-HRMS and was found to be >97%.

### 3.2. Stock and Working Solutions

Stock standard solutions of the analytes were prepared by dissolving 1 mg of each compound in 1 mL of methanol and stored at 4 °C. Working standard solutions (500 ng/mL) were prepared daily by dilution using methanol and water.

### 3.3. Instrumentation

Experiments were performed using a Triple TOF 4600 (ABSciex, Darmstadt, Germany) coupled with a micro-LC (Eksigent, Darmstadt, Germany), an autosampler set at 5 °C and a thermostated column compartment. A Halo C18 2.7 μm, 90 Å, 0.5 × 50 mm^2^ column (Eksigent, Darmstadt, Germany) was employed for chromatography at a flow rate of 55 µL/min, and the mobile phases A and B were acetonitrile/0.01% formic acid/isopropanol 80/20 *v*/*v* and water/0.01% formic acid, respectively. The following gradient was used: 5% of phase B for 0.5 min, gradually increasing to 98% in the next 7.5 min. These conditions were maintained for 0.5 min, and then the column was re-equilibrated (at the initial conditions) for 1.5 min.

Mass spectrometry was performed with an ESI source in negative-ionization mode. The resolution was 30,000 (full width at half maximum). Data acquisition mainly consisted of a full MS^1^ scan (*m*/*z* 50–850) and information-dependent acquisition (IDA)-TOF-MS/MS fragmentation events using 40 V collision energy (CE) with 15 V collision energy spread (CES) for each candidate ion in each data acquisition cycle (1091). The MS resolution working conditions were as follows: ion energy 1 (IE1) −2.3, vertical steering (VS1) −0.65, horizontal steering (HST) 1.15 and vertical steering 2 (VS2) 0.00. The use of TOF-MS and IDA-TOF-MS/MS provide both quantitation and confirmation in a single run.

For data acquisition and processing, MultiQuant 3.0.2 and PeakView 2.1 (ABSciex, Darmstadt, Germany) were used. EICs were obtained with the use of MultiQuant 3.0.2, which created the base peak chromatograms for the masses that achieved a 0.01 Da mass accuracy width. The relative tolerance of the retention time was set within a margin of ±2.5%.

### 3.4. Sample Preparation

To prepare the samples, 4 mL of methanol was added to 1 mL of milk in a screw cap glass centrifuge tube, and the mixture was vortexed for 30 s. After centrifugation (4000× *g* for 10 min), 500 μL of the supernatant was collected and mixed with 500 μL of water in a vial. This mixture was directly injected into the LC-MS/MS for analysis.

### 3.5. Method Validation

To assess the linearity and LODs and LOQs, 5–500 ng/mL solutions of 14OPA, 10OPA, 9OPA, 8OPA, 7OPA, 6OPA, 16OSA, 12OSA, 10OSA, 9OSA, 8OSA, 7OSA, 6OSA and RA (3 replicates; 9 levels (5, 10, 30, 50, 100, 200, 300, 400 and 500 ng/mL); *n* = 3 × 9) were used. LOD and LOQ were calculated using the signal-to-noise method, which is frequently applied to analytical methods that exhibit baseline noise, as discussed in the International Council for Harmonization (ICH) guidelines [28]. A signal-to-noise ratio (S/N) of 3 is generally accepted for estimating LOD, while a signal-to-noise ratio of 10 is generally accepted for estimating LOQ.

To determine the recovery and the intraday and interday variations, cow milk samples were spiked at three different concentration levels. The recovery was used for the quantification of the selected lipid components in milk. The intraday precision of the assay was estimated by calculating the relative standard deviation (RSD) for the analysis of QC samples in six replicates, and interday precision was determined by the analysis of six replicate QC samples across three consecutive days.

### 3.6. Milk Samples

Twenty different, pasteurized, whole milk brand products, commercially available in the Greek market (Athens), were analyzed with the suggested method. Eight goat milk products and 12 cow milk products were examined in the present study.

### 3.7. Statistics

Comparisons between two data groups (with *n* ≥ 3 independent experiments) were analyzed using a Student’s *t*-test. Values of <0.05 were considered significant and are indicated as * *p* < 0.05, ** *p* < 0.01 or *** *p* < 0.001.

## 4. Conclusions

In conclusion, by applying an LC-HRMS method, which involves mild sample preparation conditions, avoiding time-consuming extraction, pre-separation or derivatization procedures, 13 free SOFAs were simultaneously identified and quantified in cow and goat milk samples. This robust method permitted the rapid analysis (a single 10-min run) of milk samples, uncovering the existence of various regioisomers of OSAs and OPAs carrying the oxo functionality at different positions of the long chain. The most abundant SOFAs in both cow and goat milk were proven to be 8OSA, 9OSA, 7OSA, 10OSA and 10OPA, while higher contents of SOFAs were found in cow milk in comparison to goat milk. A “suspect” HRMS analysis approach revealed the presence of RA, which is isobaric to OSA, in all samples in much higher quantities than that of each particular SOFA. This unsaturated fatty acid, which is the main component of castor oil triglycerides (>90%), was found at mean content values of 534.3 ± 6.0 μg/mL and 460 ± 8.1 μg/mL in cow and goat fresh milk samples, respectively.

## Figures and Tables

**Table 1:** Oxo fatty acid standards used in the chromatographic method.

Compound	Abbreviation	Structure	Theoretical Mass [M − H]^−^	Mass Error (ppm)	Retention Time (min)
14-Oxopalmitic acid	14OPA	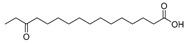	269.2122	0.74	4.47
10-Oxopalmitic acid	10OPA	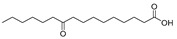	269.2122	0.85	4.60
9-Oxopalmitic acid	9OPA	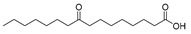	269.2122	0.80	4.66
8-Oxopalmitic acid	8OPA	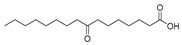	269.2122	0.69	4.73
7-Oxopalmitic acid	7OPA	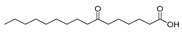	269.2122	0.75	4.81
6-Oxopalmitic acid	6OPA	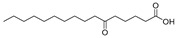	269.2122	0.82	4.93
16-Oxostearic acid	16OSA	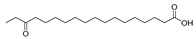	297.2435	1.01	5.11
12-Oxostearic acid	12OSA	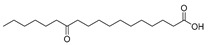	297.2435	1.11	5.19
10-Oxostearic acid	10OSA	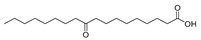	297.2435	0.92	5.28
9-Oxostearic acid	9OSA	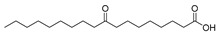	297.2435	0.95	5.34
8-Oxostearic acid	8OSA	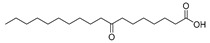	297.2435	1.09	5.41
7-Oxostearic acid	7OSA	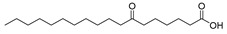	297.2435	1.10	5.49
6-Oxostearic acid	6OSA	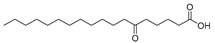	297.2435	1.03	5.60

**Table 2:** Calibration curve data and limits of detection (LOD) and quantification (LOQ) (range 5–500 ng/mL).

Analyte	Linearity (R^2^)	LOD (ng/mL)	LOQ (ng/mL)
14OPA	0.990	0.3	1.0
10OPA	0.993	0.5	1.4
9OPA	0.995	0.3	1.0
8OPA	0.992	0.5	1.4
7OPA	0.993	0.5	1.4
6OPA	0.990	0.5	1.4
16OSA	0.991	0.8	2.4
12OSA	0.990	0.5	1.4
10OSA	0.992	0.5	1.4
9OSA	0.995	0.5	1.4
8OSA	0.991	0.5	1.4
7OSA	0.991	0.8	2.4
6OSA	0.990	0.8	2.4
RA	0.996	0.6	2.0

**Table 3:** Accuracy (recovery %), matrix factor (MF) values and precision data (RSD, %) in spiked milk samples.

Analyte	Spike Level (ng/mL)	Recovery (%)	MF	RSD_r_ (%)	RSD_R_ (%)
14OPA	50	79.3	0.7	5.3	7.3
	200	82.3	0.8	0.7	5.8
	500	81.7	0.9	5.9	6.3
10OPA	50	82.9	0.7	7.9	10.1
	200	99.5	1.0	6.1	11.3
	500	83.1	0.9	1.6	11.5
9OPA	50	79.1	0.7	4.4	3.7
	200	86.7	0.9	6.4	5.1
	500	84.1	0.7	0.9	2.3
8OPA	50	79.1	0.6	3.7	2.1
	200	82.4	0.9	0.5	1.1
	500	84.7	0.8	2.7	0.8
7OPA	50	77.0	0.7	9.3	11.1
	200	75.9	0.9	7.2	7.7
	500	87.7	0.7	5.0	10.3
6OPA	50	73.4	0.7	5.9	4.9
	200	73.9	0.8	7.2	6.1
	500	82.4	0.7	1.1	6.5
16OSA	50	81.2	0.7	7.2	10.4
	200	90.9	0.9	3.2	8.3
	500	112.0	1.0	0.1	6.9
12OSA	50	83.3	0.7	4.3	1.9
	200	84.2	0.8	2.1	4.9
	500	102.9	1.0	1.7	2.0
10OSA	50	76.3	0.8	7.3	11.1
	200	81.9	0.9	7.7	3.9
	500	87.5	0.8	5.3	7.4
9OSA	50	78.4	0.7	2.5	10.1
	200	74.5	0.7	6.8	10.3
	500	96.2	0.9	1.2	11.2
8OSA	50	74.2	0.7	5.8	4.4
	200	83.4	0.7	2.1	5.5
	500	94.9	1.1	2.2	1.0
7OSA	50	81.6	0.7	3.5	10.3
	200	81.5	0.8	6.8	10.8
	500	91.3	1.1	3.2	11.1
6OSA	50	84.0	0.7	3.3	7.2
	200	95.4	0.9	4.8	6.4
	500	96.1	1.1	1.3	8.2
RA	50	90.1	1.0	6.6	1.5
	200	89.7	0.8	3.2	4.1
	500	99.2	0.9	4.1	3.0

MF: matrix factor; RSD_r_: intraday relative standard deviation; RSD_R_: interday relative standard deviation.

**Table 4:** Contents of free saturated oxo fatty acids (SOFAs) and ricinoleic acid (RA) in cow milk and goat milk samples (μg/mL fresh milk).

	Cow Milk (*n* = 12), Triplicates				Goat Milk (*n* = 8), Triplicates			
SOFAs and RA	Minimum Value (μg/mL)	Maximum Value (μg/mL)	Mean Value ± SD (μg/mL)	Level of Significance	Minimum Value (μg/mL)	Maximum Value (μg/mL)	Mean Value ± SD (μg/mL)	Level of Significance
14OPA	8.1	32.4	22.3 ± 4.0	***	6.6	23.7	15.4 ± 3.1	***
10OPA	31.7	127.5	76.7 ± 7.2	***	<LOQ ^e^	84.6	41.9 ± 5.6	**
9OPA	9.0	55.4	23.3 ± 5.6	***	<LOQ ^e^	37.8	17.3 ± 4.1	**
8OPA	6.4	48.5	14.7 ± 3.1	***	<LOQ ^e^	18.5	9.4 ± 1.6	***
7OPA	<LOQ ^a^	111.6	43.6 ± 5.2	**	<LOQ ^e^	20.3	15.0 ± 3.1	**
6OPA	<LOQ ^b^	52.3	12.5 ± 4.1	**	4.4	23.1	1.6 ± 2.9	**
16OSA	<LOQ ^c^	30.3	6.5 ± 6.1	**	<LOQ	<LOQ	-	-
12OSA	<LOQ ^d^	59.9	17.4 ± 5.1	**	<LOQ ^a^	18.0	6.8 ± 5.3	*
10OSA	<LOQ ^e^	118.3	47.6 ± 4.6	***	<LOQ ^a^	53.8	20.3 ± 5.1	*
9OSA	26.9	176.8	89.0 ± 6.5	***	46.6	85.3	66.9 ± 7.1	***
8OSA	<LOQ ^b^	239.5	96.9 ± 7.2	**	<LOQ ^a^	114.1	45.6 ± 8.0	*
7OSA	<LOQ ^b^	120.6	66.8 ± 6.5	**	<LOQ ^a^	101.2	42.1 ± 7.2	*
6OSA	<LOQ	<LOQ	-	-	<LOQ	<LOQ	-	-
RA	181.7	620.2	534.3 ± 6.0	***	231.1	411.2	460.4 ± 8.1	***

Content lower than LOQ in ^a^4, ^b^5, ^c^9, ^d^6, ^e^1 samples; the mean value was determined using medium-bound approach; SD: standard deviation; * *p* < 0.05, ** *p* < 0.01, *** *p* < 0.001.

## Data Availability

The data presented in this study are available on request from the corresponding author. The data are not publicly available due to privacy.

## Supplementary Materials

The following are available online at https://www.mdpi.com/2218-1989/11/1/46/s1, Figure S1: MS/MS spectra of saturated oxo fatty acids: (a) 14OPA, (b) 10OPA, (c) 9OPA), (d) 8OPA, (e) 7OPA, (f) 6OPA, (g) 16OSA, (h) 12OSA, (i) 10OSA, (j) 9OSA, (k) 8OSA, (l) 7OSA and (m) 6OSA.
