# Supplementary material for: Free Saturated Oxo Fatty Acids (SOFAs) and Ricinoleic Acid in Milk Determined by a Liquid Chromatography-High-Resolution Mass Spectrometry (LC-HRMS) Method
Source: Metabolites. 2021 Jan 11;11(1):46. doi: 10.3390/metabo11010046 (PMC7828063; doi:10.3390/metabo11010046)
Supplement: Supplementary file 1 [file metabolites-11-00046-s001.pdf]

Free Saturated Oxo Fatty Acids (SOFAs) and Ricinoleic Acid in Milk Determined by a Liquid Chromatography-High Resolution Mass Spectrometry (LC-HRMS) Method

Maroula G. Kokotou, Charikleia S. Batsika, Christiana Mantzourani and George Kokotos*

Department of Chemistry, National and Kapodistrian University of Athens, Athens 15771, Greece

SUPPLEMENTARY MATERIAL

**Table of Contents**	**Page**
**Figure S1.** MS/MS spectra of saturated oxo fatty acids: (a) 14OPA, (b) 10OPA, (c) 9OPA), (d) 8OPA, (e) 7OPA, (f) 6OPA, (g) 16OSA, (h) 12OSA, (i) 10OSA, (j) 9OSA, (k) 8OSA, (l) 7OSA, (m) 6OSA.	**S2**

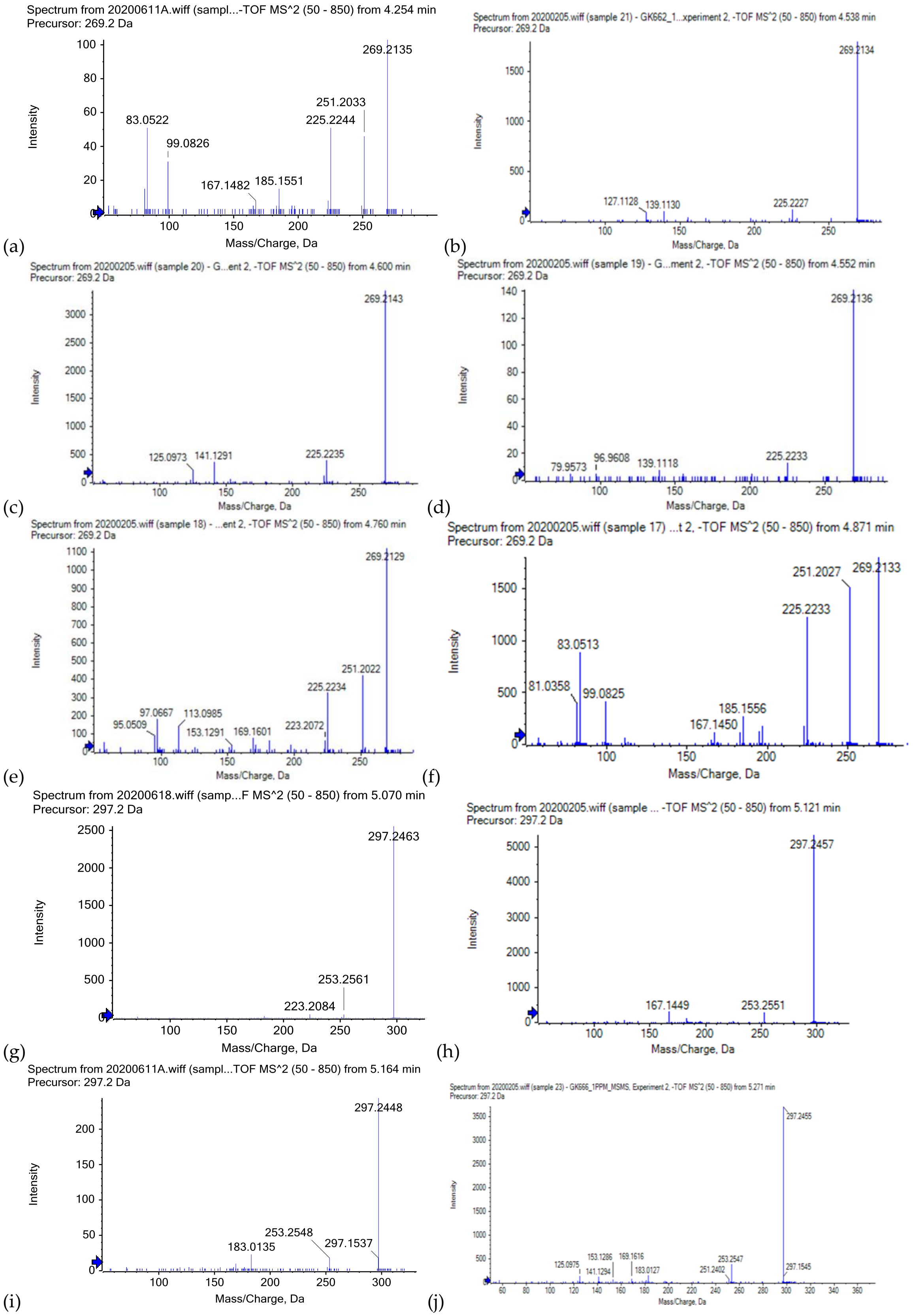

Spectrum from 20200611A.wiff (sampl...-TOF MS^2 (50 - 850) from 4.254 min
Precursor: 269.2 Da
269.2135
251.2033
225.2244
83.0522
99.0826
167.1482
185.1551
Intensity
Mass/Charge, Da
(a)
Spectrum from 20200205.wiff (sample 21) - GK662_1...xperiment 2, -TOF MS^2 (50 - 850) from 4.538 min
Precursor: 269.2 Da
269.2134
127.1128
139.1130
225.2227
Intensity
Mass/Charge, Da
(b)
Spectrum from 20200205.wiff (sample 20) - G...ent 2, -TOF MS^2 (50 - 850) from 4.600 min
Precursor: 269.2 Da
269.2143
125.0973
141.1291
225.2235
Intensity
Mass/Charge, Da
(c)
Spectrum from 20200205.wiff (sample 19) - G...ment 2, -TOF MS^2 (50 - 850) from 4.552 min
Precursor: 269.2 Da
269.2136
79.9573
96.9608
139.1118
225.2233
Intensity
Mass/Charge, Da
(d)
Spectrum from 20200205.wiff (sample 18) - ...ent 2, -TOF MS^2 (50 - 850) from 4.760 min
Precursor: 269.2 Da
269.2129
251.2022
225.2234
223.2072
97.0667
113.0985
95.0509
153.1291
169.1601
Intensity
Mass/Charge, Da
(e)
Spectrum from 20200205.wiff (sample 17) ...t 2, -TOF MS^2 (50 - 850) from 4.871 min
Precursor: 269.2 Da
251.2027
269.2133
225.2233
83.0513
81.0358
99.0825
185.1556
167.1450
Intensity
Mass/Charge, Da
(f)
Spectrum from 20200618.wiff (samp...F MS^2 (50 - 850) from 5.070 min
Precursor: 297.2 Da
297.2463
253.2561
223.2084
Intensity
Mass/Charge, Da
(g)
Spectrum from 20200205.wiff (sample ... -TOF MS^2 (50 - 850) from 5.121 min
Precursor: 297.2 Da
297.2457
167.1449
253.2551
Intensity
Mass/Charge, Da
(h)
Spectrum from 20200611A.wiff (sampl...TOF MS^2 (50 - 850) from 5.164 min
Precursor: 297.2 Da
297.2448
253.2548
183.0135
297.1537
Intensity
Mass/Charge, Da
(i)
Mass/Charge, Da
(j)

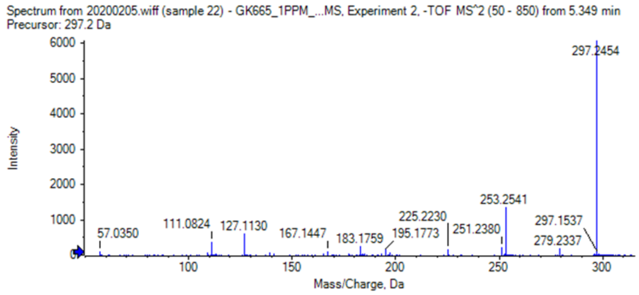

(k)

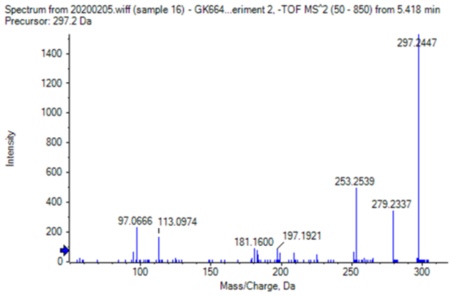

(l)

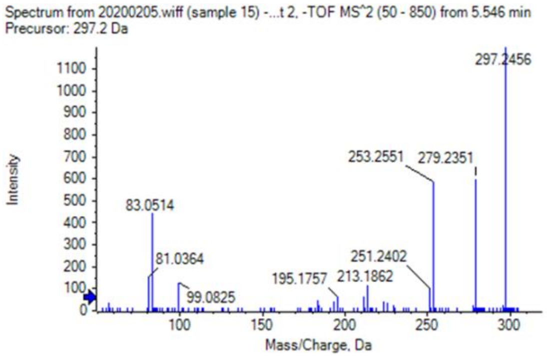

(m)

Figure S1. MS/MS spectra of saturated oxo fatty acids: (a) 14OPA, (b) 10OPA, (c) 9OPA), (d) 8OPA, (e) 7OPA, (f) 6OPA, (g) 16OSA, (h) 12OSA, (i) 10OSA, (j) 9OSA, (k) 8OSA, (l) 7OSA, (m) 6OSA.
